# The genetic association between osteoprotegerin (OPG) gene polymorphisms and bone mineral density (BMD) in postmenopausal women: A meta-analysis: Erratum

**DOI:** 10.1097/MD.0000000000014926

**Published:** 2019-03-15

In the article, “The genetic association between osteoprotegerin (OPG) gene polymorphisms and bone mineral density (BMD) in postmenopausal women: A meta-analysis”,^[1]^ which appeared in Volume 97, Issue 51 of *Medicine*, the affiliations for the authors are appearing incorrectly. They should be:

^a^Department of Orthopedics, Changshu First People's Hospital, Changshu, 215500, People's Republic of China.

^b^Department of Orthopedics, The First Affiliated Hospital of Suzhou University, Soochow 215000, People's Republic of China.
